# Lectures on Mental Disease

**Published:** 1885-03

**Authors:** 


*Lectures on Mental Disease*. By W. H. O. SANKEY, M.D. Lond., F.R.C.P., &c., &c. Second Edition, with illustrations. London: H. K. LEWIS, 136 Gower Street, W.C. 1884.

After a lapse of some years, Dr. W. H. O. Sankey has issued a second edition of his *Lectures on Mental Disease*. In its former state it was valued for its clinical utility, and has now been brought more nearly up to date, and embellished with a few well-executed chromo-lithographs, illustrating some points in the histology of the brain both in health and under conditions of mental disease. The whole book will be found to well repay perusal by those who are not specialists, and still more, of course, by those who are alienists.

The chapters on General Paralysis we thought specially good and instructive; the apparent completeness of the remissions in that disease deceives not only the patient and the patient's friends, but occasionally the doctor himself also, who is duly taunted with the incorrectness of his prognosis, and, being perhaps wrongly convinced of his own error, proceeds to eat the leek in meek submission, until the relapse and speedy death justify his original verdict. The simple yet scientific classification of mental diseases which Dr. Sankey adopts is much to be commended and preferred over either Griesinger's purely symptomatic arrangement or the hair-splitting mode of sub-division practised by Skae.

The book hardly lays claim to be a perfect treatise on insanity, of which for English readers there is still an absence. When will some one "supply a want which has long been felt," and give us a complete text-book, without which "no physician's library would be complete"? Dr. Savage's recent manual is very good *quantum valeat*, but that is not *quantum sufficit*. To our leading alienists the whole profession, like the daughters of the horse-leech, is crying, "Give, give!"

